# An Integrated Model Combining Machine Learning and Deep Learning Algorithms for Classification of Rupture Status of IAs

**DOI:** 10.3389/fneur.2022.868395

**Published:** 2022-05-12

**Authors:** Rong Chen, Xiao Mo, Zhenpeng Chen, Pujie Feng, Haiyun Li

**Affiliations:** Beijing Key Laboratory of Fundamental Research on Biomechanics in Clinical Application, School of Biomedical Engineering, Capital Medical University, Beijing, China

**Keywords:** intracranial aneurysm, rupture risk, deep learning, machine learning, hemodynamic cloud

## Abstract

**Background:**

The rupture risk assessment of intracranial aneurysms (IAs) is clinically relevant. How to accurately assess the rupture risk of IAs remains a challenge in clinical decision-making.

**Purpose:**

We aim to build an integrated model to improve the assessment of the rupture risk of IAs.

**Materials and Methods:**

A total of 148 (39 ruptured and 109 unruptured) IA subjects were retrospectively computed with computational fluid dynamics (CFDs), and the integrated models were proposed by combining machine learning (ML) and deep learning (DL) algorithms. ML algorithms that include random forest (RF), k-nearest neighbor (KNN), XGBoost (XGB), support vector machine (SVM), and LightGBM were, respectively, adopted to classify ruptured and unruptured IAs. A Pointnet DL algorithm was applied to extract hemodynamic cloud features from the hemodynamic clouds obtained from CFD. Morphological variables and hemodynamic parameters along with the extracted hemodynamic cloud features were acted as the inputs to the classification models. The classification results with and without hemodynamic cloud features are computed and compared.

**Results:**

Without consideration of hemodynamic cloud features, the classification accuracy of RF, KNN, XGB, SVM, and LightGBM was 0.824, 0.759, 0.839, 0.860, and 0.829, respectively, and the AUCs of them were 0.897, 0.584, 0.892, 0.925, and 0.890, respectively. With the consideration of hemodynamic cloud features, the accuracy successively increased to 0.908, 0.873, 0.900, 0.926, and 0.917. Meanwhile, the AUCs reached 0.952, 0.881, 0.950, 0.969, and 0.965 eventually. Adding consideration of hemodynamic cloud features, the SVM could perform best with the highest accuracy of 0.926 and AUC of 0.969, respectively.

**Conclusion:**

The integrated model combining ML and DL algorithms could improve the classification of IAs. Adding consideration of hemodynamic cloud features could bring more accurate classification, and hemodynamic cloud features were important for the discrimination of ruptured IAs.

## Introduction

Intracranial aneurysms (IAs) are pathological dilations at major branching of the Willis circle, occurring in 3–5% of the population (1). The rupture of IAs would lead to subarachnoid hemorrhage (SAH) with high mortality and disability rates (2). How to accurately assess the rupture risk of IAs remains a challenge. Hemodynamic is considered to play a crucial role in the growth and rupture of IAs. Many studies attempt to utilize their morphological variables and hemodynamic parameters to assess the rupture risk of IAs (3–6).

The morphological variables related to the rupture risk of IAs included location, height, aspect ratio (AR), size ratio (SR), size, and so on. Additionally, the hemodynamic parameters associated with the rupture risk of IAs contained wall shear stress (WSS), oscillatory shear index (OSI), pressure and low shear area (LSA), and so on. Duan et al. (7) applied AR, SR, location, and other morphological variables combining age, gender, and other clinical risk factors to assess the rupture risk of IAs with multivariate logistic regression model and further evaluated the importance of morphological variables with the univariate analysis method. Juchler et al. (8) explored the irregular shape of IAs and found that the irregular shape was related to the stability of vascular wall, and that the size as well as location of IAs had a great impact on their rupture. Regarding the hemodynamic parameters, Cebral et al. (5) utilized max WSS, LSA, concentrated inflow streams, and some other hemodynamic parameters to compare the differences between ruptured and unruptured IAs using the Student's *t*-test. In addition, Xu et al. (9) investigated the influence of blood flow instability on the rupture of IAs and explored the discrepancy of WSS, LSA, and pressure loss coefficient in ruptured and unruptured IAs statistically.

At present, machine learning (ML) has shown significant potential in the assessment of rupture risk of IAs (10–13). Liu et al. (11) fed some morphological features extracted from PyRadimics and clinical features into general linear, ridge, and lasso regression models to predict aneurysm stability and obtained the highest area under the curve (AUC) of 0.86. Ou et al. (12) applied SVM, artificial neural network (ANN), XGBoost, and logistic regression algorithms on multidimensional data of morphologies, demographics, clinical features, lifestyle behaviors, and lipid profiles to assess rupture risk of IAs and achieved the best AUC value of 0.882 with XGBoost. Detmer et al. (13) calculated morphological variables and hemodynamic parameters and utilized logistic group lasso regression modeling to discriminate the ruptured from unruptured IAs, and the AUC of the model was 0.8359. Shi et al. (14) applied the logistic regression (LR), SVM, RF, and multilayer perceptron (MLP) algorithms to hemodynamic parameters derived from CFD such as WSS, OSI, and pressure to assess the rupture risk of IAs, and SVM achieved the best AUC of 0.88. Tanioka et al. (15) employed three RF models to analyze the effects of maximum size, projection length, neck width, WSS, OSI, flow velocity, and other morphological variables and hemodynamic parameters on the classification of the rupture status of IAs, and three models' accuracy was 0.77, 0.71, and 0.78, respectively. In addition, Silva et al. (16) adopted RF and SVMs with linear and RBF kernel to distinguish the ruptured and unruptured IAs based on aneurysm size, location, aneurysm side, age, sex, and other history information, with the AUCs reached 0.81, 0.77, and 0.78. In addition, deep learning was also beginning to be applied to the risk assessment of IAs. Kim et al. (17) took advantage of a convolutional neural network (CNN) to 3D digital subtraction angiography for rupture risk assessment in small-sized IAs and showed an accuracy of 0.77 and AUC of 0.76. Liu et al. (18) applied a feed-forward artificial neural network to morphological features, demographic factors, and hypertension and smoking histories for the assessment of rupture risk of communicating artery aneurysms, achieving the highest AUC of 0.95. Bizjak et al. (19) applied univariate thresholding, multivariate random forest and multilayer perceptron (MLP) learning, and deep shape learning on morphological features and deep shape features to predict IA growth. The deep shape learning method could achieve the highest accuracy of 0.82. Yang et al. (20) utilized CNN on hemodynamic factors of WSS and strain to predict the rupture risk of cerebral aneurysms, and the best AUC was up to 0.883.

The accuracy of the assessment of rupture risk of IAs still needs to be improved for clinical decision-making. Current studies mainly considered the morphological variables and hemodynamic parameters, neglecting the hemodynamic cloud features reflected the spatial distribution characteristics of hemodynamic, as well as the features of impingement zone and inflow jet. It is generally accepted that complex flow patterns, small impingement regions, and narrow inflow jet are more likely to be identified in ruptured IAs (21). We speculate that hemodynamic clouds and impingement zone and inflow jet would be helpful to assess the rupture risk of IAs. In this paper, we proposed an integrated model combining ML and DL algorithms to classify ruptured and unruptured IAs, adopting the morphological variables, hemodynamic parameters, and hemodynamic clouds within the impingement zone and inflow jet.

## Method

**Figure 1:**
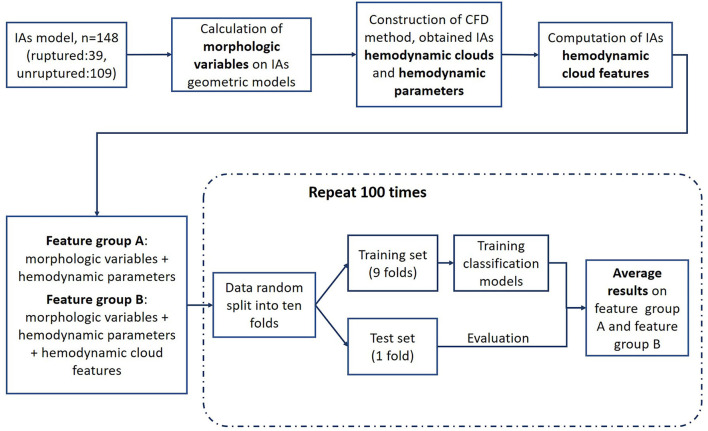
Framework of the integrated model.

In this paper, we proposed an integrated model combining machine learning (ML) and deep learning (DL) algorithms. ML algorithms were applied to classify the ruptured and unruptured IAs, which included random forest (RF), k-nearest neighbor (KNN), XGBoost (XGB), support vector machine (SVM), and LightGBM. In addition, a Pointnet DL algorithm was used to extract the hemodynamic cloud features for the ML classification models. The morphological variables were computed on the IA geometric models, and the hemodynamic clouds were obtained from computational fluid dynamic (CFD), and hemodynamic parameters were calculated on hemodynamic clouds. In particular, the hemodynamic clouds within impingement zone and inflow jet were determined and served as the inputs to the DL algorithm for features extraction, and the extracted hemodynamic cloud features were acted as the inputs to the classification models. Based on morphological variables and hemodynamic parameters with/without hemodynamic cloud features, the rupture status of IAs was classified by these ML algorithms' classification models. The framework of the integrated model is shown in Figure 1.

### Geometric Model and Morphological Variables

**Figure 2:**
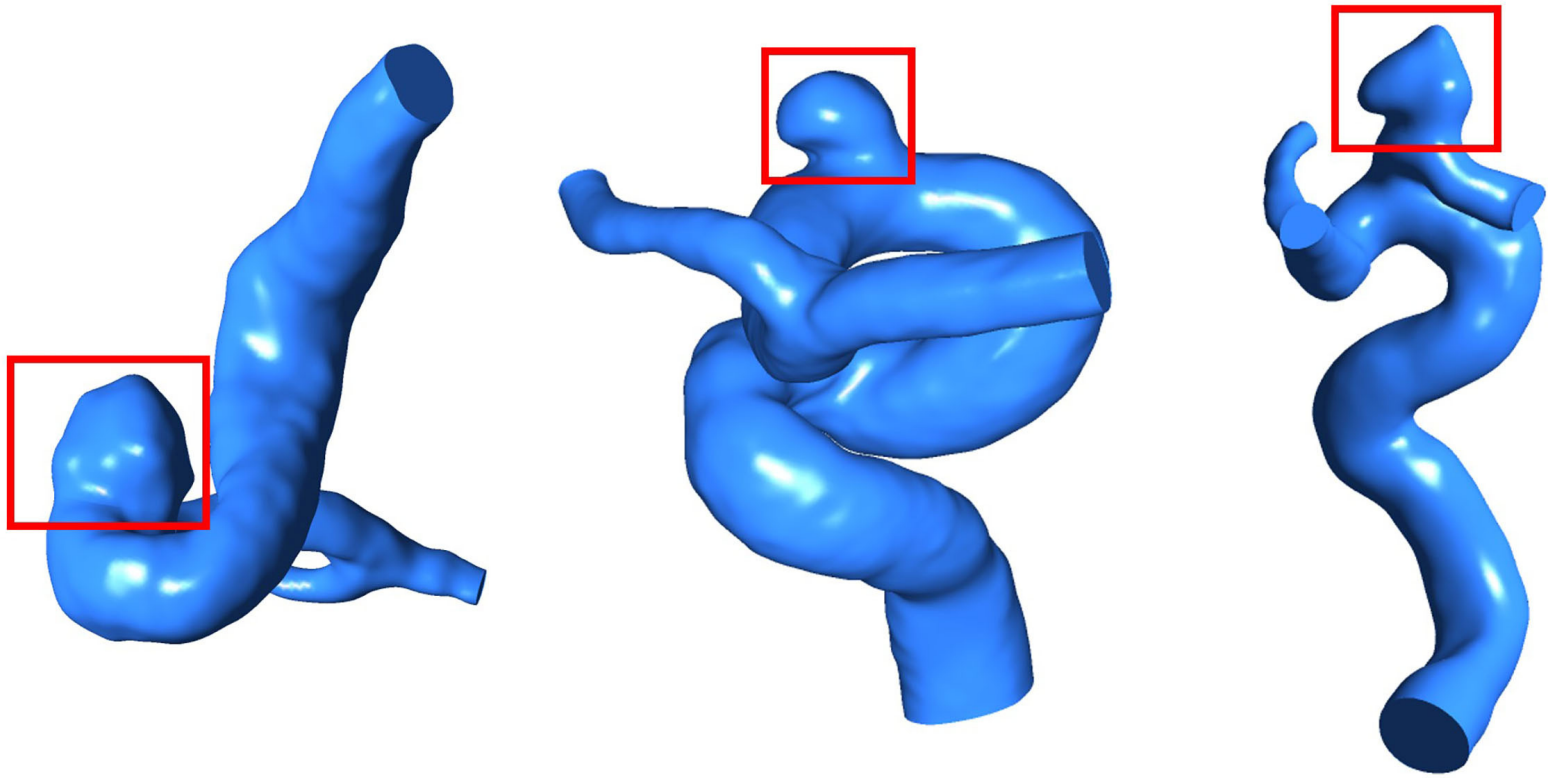
IA models (the areas marked by red boxes are sacs of IAs).

A total of 148 IAs located at the internal carotid artery were recruited, including 109 unruptured and 39 ruptured IAs. All the participants provided their informed consent. Besides, the protocol of this study was approved by the Ethics Committee of Beijing Tiantan Hospital Affiliated to Capital Medical University. The 3D rotational angiography images of each IAs were acquired using a GE LCV + Digital Subtraction system (LCV; GE Medical Systems), and then, the 88 projection images were transferred to a 3D dataset using isotropic voxels on a dedicated GE workstation (Advantage Unix; GE Medical Systems). Subsequently, the IA geometry was extracted from the acquired raw Digital Imaging and Communications in Medicine (DICOM) files with a commercial software Mimics 10.0 (Belgium Materialize Company), next, the IA geometry was converted into a triangulated surface model, and the blood vessel wall was built based on the surface along the normal direction of the wall. Finally, the established model was modified as a 3D-solid volume model upon software SolidWorks 2012 (SolidWorks Corp, Concord, MA) (Figure 2).

Applying the IA geometric model, the neck width, height, diameter of vessel, size ratio (SR), and aspect ratio (AR) were determined by Geomagic Studio 2013 (Raindrop Geomagic, Durham, USA). Surface area, volume, and surface area/volume (S/V) of IAs were calculated by CFD-Post. Besides, the geometric shapes of IAs were classified into a smooth type and an irregular type with daughter sacs. In addition, in terms of the location, aneurysms were classified into a side wall type or a bifurcated aneurysm. So, 10 morphological variables were achieved.

### Hemodynamic Clouds and Hemodynamic Parameters

The hemodynamic clouds and the hemodynamic parameters were achieved based on CFD with ANSYS 18.0 (ANSYS Inc., Canonsburg, PA, USA). First, the computational mesh was generated with ANSYS ICEM 18.0, by setting the mesh element size to 0.25 mm, and we generated boundary fitted prism layers with an average node space increasing by a ratio of 1.3 as well. Then, we applied ANSYS Fluent 18.0 to solve the flow-governing Navier–Stokes equation, assuming the aneurysm wall to be no-slip and rigid and modeling the blood as incompressible under laminar flow conditions, whereas the density and dynamic viscosity were set to 1,060 kg/m^3^ and 0.004 N·s/m^2^, respectively. The inlet boundary conditions included a pulsatile velocity that was determined by a transcranial Doppler (TCD) scanning from a healthy subject, and a zero-pressure gradient was adopted at the outlets. The cardiac cycle was 0.8 s with a time step of 0.01 s for each cardiac cycle (22). A total of two cycles were simulated whereas the systolic phase of the second cardiac cycle was chosen for output. Finally, the hemodynamic clouds were achieved, from which the hemodynamic parameters were calculated.

**Figure 3:**
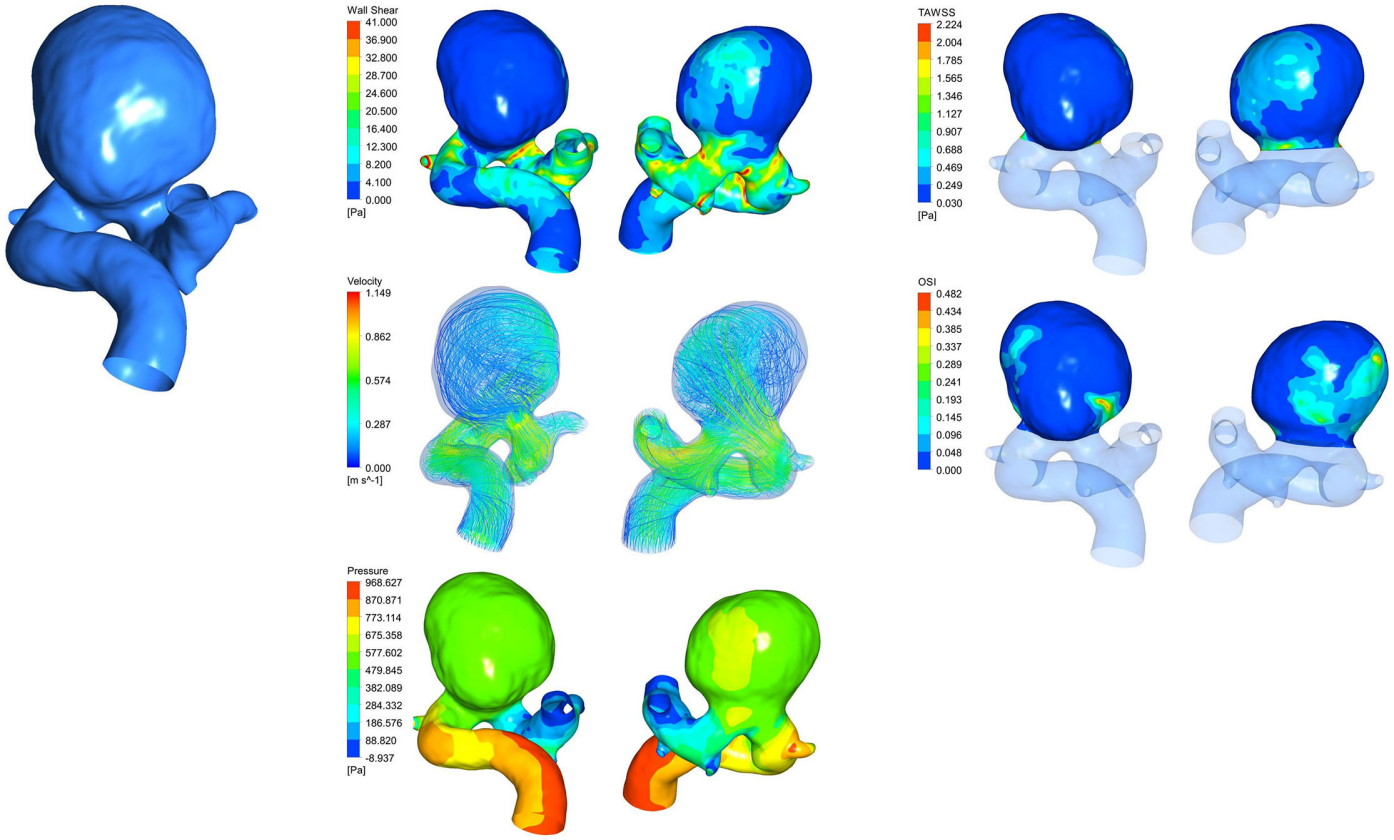
Hemodynamic clouds of WSS, TAWSS, velocity, OSI, and pressure.

A total of five acquired hemodynamic clouds of WSS, OSI, pressure, velocity, and time average WSS (TAWSS) are shown in Figure 3, and the hemodynamic cloud features were extracted by the DL algorithm.

**Figure 4:**
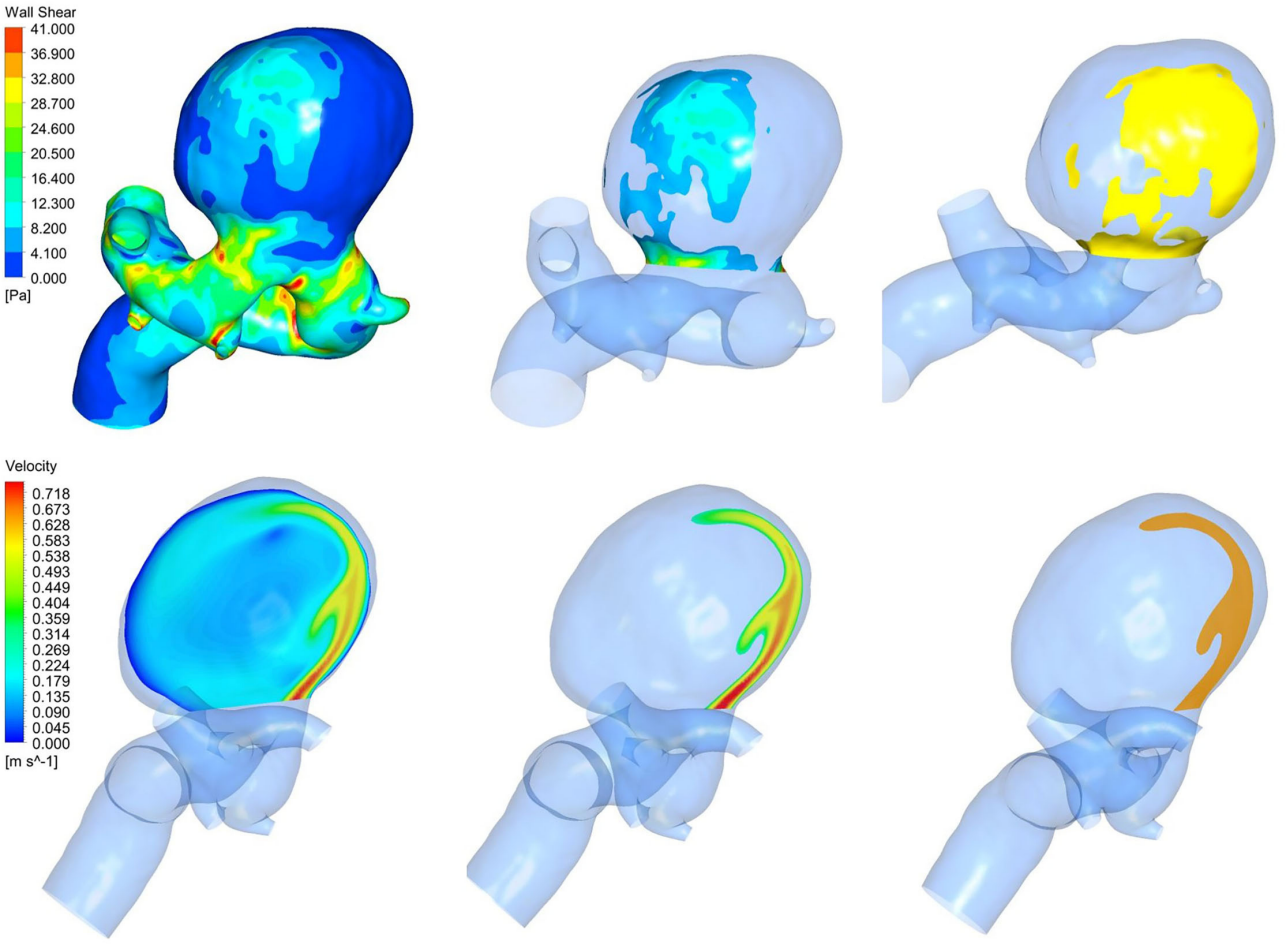
The impingement zone (yellow) and the inflow jet (orange).

After obtaining the hemodynamic clouds with CFD, we adopted the hemodynamic clouds within impingement zone and inflow jet as the inputs to the DL algorithm for feature extraction. To determine impingement zone and inflow jet in the sac of IAs, the profiles of WSS and blood velocity were computed. Inflow jet was determined by referring to the direction and magnitude of blood flow inside the sac (Figure 4), and the profile with high velocity in the streamline was regarded as inflow jet. Impingement zone was determined by referring to the region with high WSS (>80% of the maximum WSS in sac) distribution and the influence area of the inflow jet, as shown in Figure 4. Finally, the hemodynamic cloud features were extracted from the hemodynamic clouds within impingement zone and inflow jet applying a Qi et al. (23) DL algorithm. The extracted hemodynamic cloud features along with morphological variables and hemodynamic parameters were acted as the inputs to the classification models.

**Figure 5:**
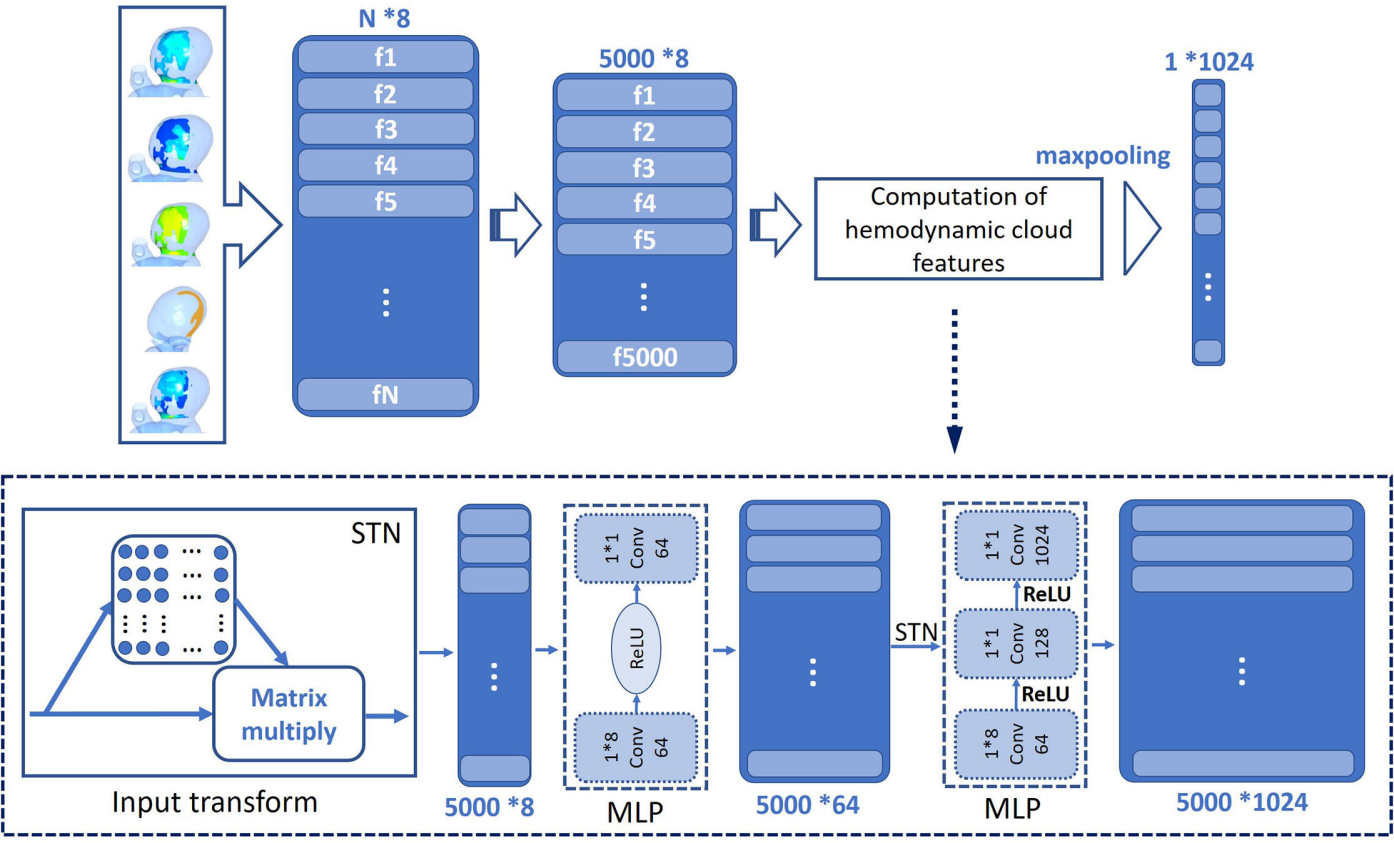
Framework of DL algorithm.

The data exported from hemodynamic clouds were point cloud. Combing five acquired hemodynamic clouds of WSS, OSI, pressure, velocity, and time average WSS (TAWSS), each point cloud contains not only a three-dimensional coordinate value (x, y, z), but also the values of WSS, OSI, pressure, velocity, and TAWSS. The DL algorithm was established based on Pointnet (23) to directly process the point cloud data, and the algorithm framework is shown in Figure 5.

In this study, 5,000 point clouds were randomly chosen from the hemodynamic clouds within impingement zone and inflow jet as the inputs to the DL algorithm, and a T-net was utilized to convert the point cloud data. A shared MLP was applied for feature extraction, transforming the data matrix to a feature matrix and increasing the dimension from 8 to 64 meanwhile. Additionally, the T-net was utilized for feature conversion the second time. The purpose of using T-net two times is to predict an affine transformation matrix to align input points and point features in the sample, so as to improve the network performance. Subsequently, feature extraction was carried out again using another shared MLP and the dimension of feature matrix increased from 64 to 1,024 gradually. The dimension of data is increased from 8 to 1,024 to avoid the loss of important features when maxpooling is finally used. Maxpooling is a symmetric function that aggregates the features of all points at the last layer of the network and outputs a 1,024-dimensional feature, which is defined as the hemodynamic cloud feature.

For hemodynamic parameters, high OSI area (HOA), LSA, WSS, OSI, pressure, and velocity and some corresponding parameters such as maximum, average, and minimum values of them were calculated by CFD-Post. Energy loss (EL) of IAs was also computed, which has been described in detail in our previous study (24). A total of eighteen hemodynamic parameters were ultimately used for classification.

### Machine Learning Algorithms

**Figure 6:**
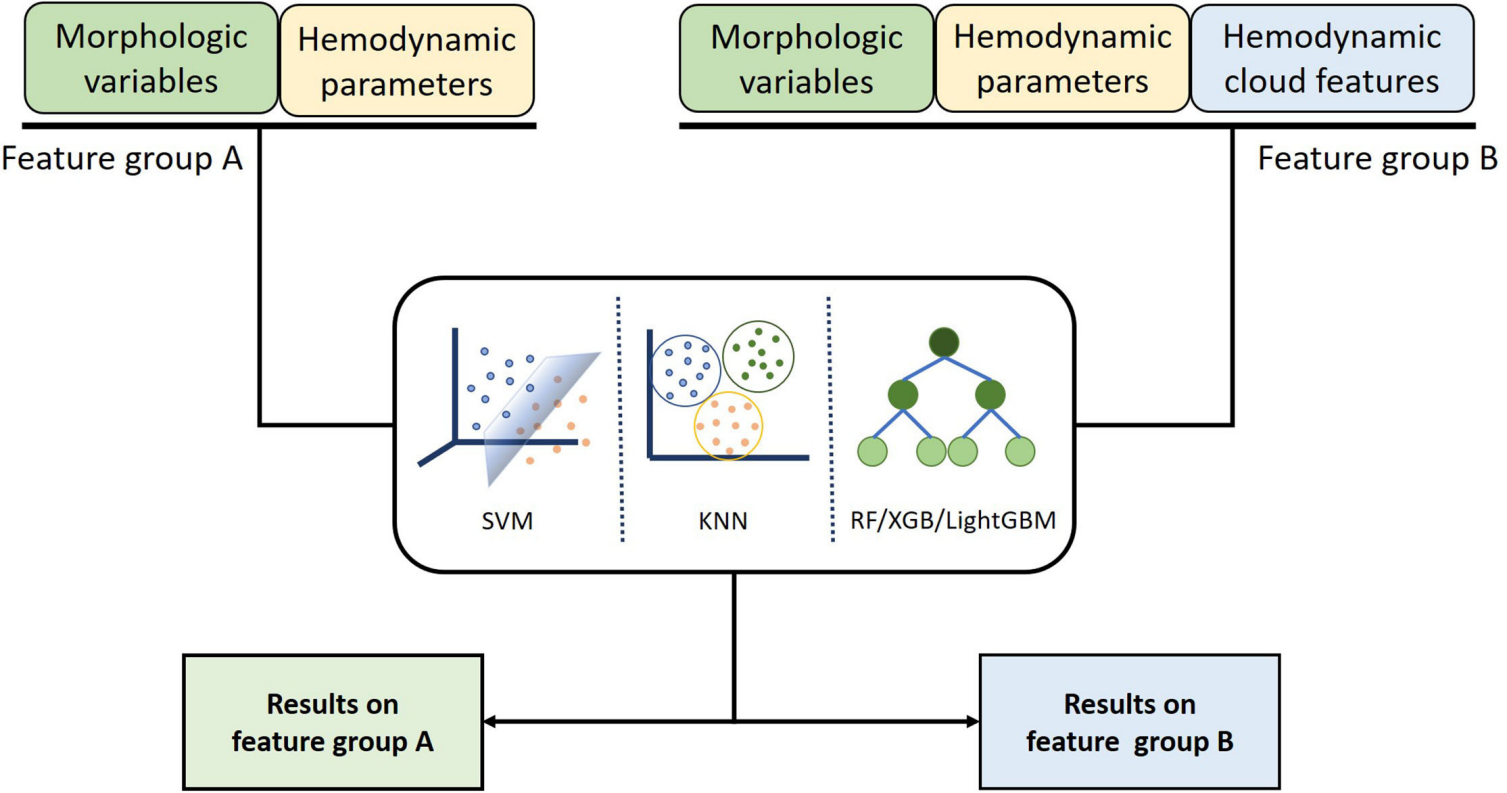
Classification models inputting different features were established using machine learning algorithm to compare the influence of different features on classification results.

To distinguish the ruptured IAs from the unruptured, five ML algorithms that include RF, KNN, XGB, SVM, and LightGBM were served as classification models. The input data comprise morphological variables, hemodynamic parameters, and hemodynamic cloud features and are divided into two groups, feature group A includes morphological variables and hemodynamic parameters, and feature group B contains morphological variables, hemodynamic parameters, and hemodynamic cloud features (Figure 6). To alleviate the impact of redundant features on the classification accuracy, data in features group B were preprocessed with normalization, feature screening, and feature dimension reduction.

In feature selection, the recursive feature elimination (RFE) method (25) was applied to screen out important variables. In addition, the kernel principal component analysis (KPCA) was utilized to reduce dimension (26).

As shown in Figure 1, we adopted 10-fold cross-validation to avoid overfitting. Moreover, we repeated the cross-validation 100 times to ensure randomness of the dataset partitioning, shuffling the dataset randomly before partitioning each time. In the process of repeated cross-validation, the hyperparameters of the machine learning algorithm were always consistent, so as to ensure the validity of the results. The results of each test included accuracy, AUC, sensitivity, and specificity, and the average value of each parameter would be calculated to evaluate the model performance.

## Result

Applying the classification models to distinguish the ruptured IAs from the unruptured IAs, the performance metrics for classification was achieved. In our model, applying the ML algorithms to morphological variables and hemodynamic parameters, the classification accuracy of RF, KNN, XGB, SVM, and LightGBM was 0.824, 0.759, 0.839, 0.860, and 0.829, respectively, and the AUCs of them were 0.897, 0.584, 0.892, 0.925, and 0.890. Adding consideration of hemodynamic cloud features, the accuracy successively increased to 0.908, 0.873, 0.900, 0.926, and 0.917, respectively. Meanwhile, the AUCs reached 0.952, 0.881, 0.950, 0.969, and 0.965 eventually.

**Figure 7:**
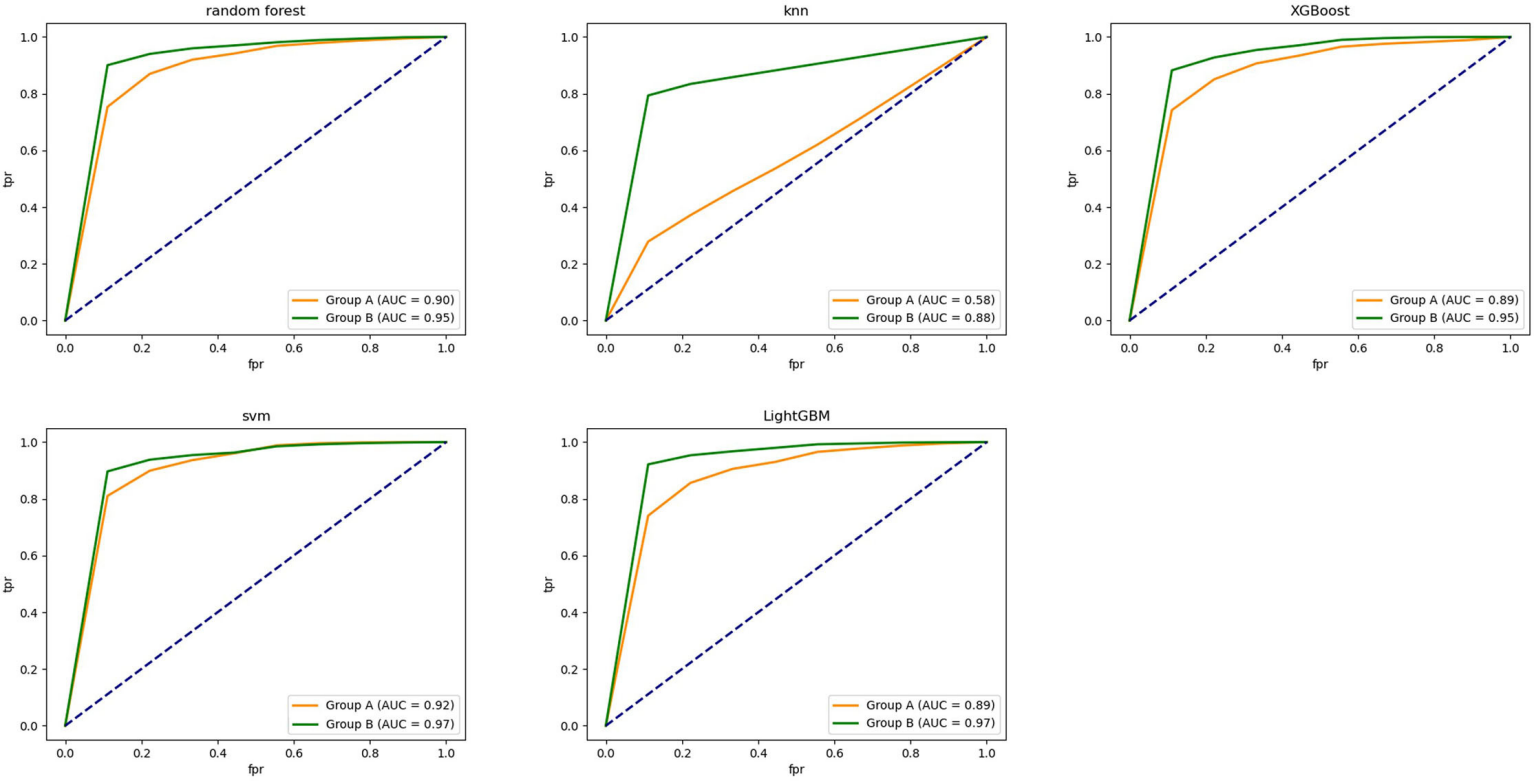
The ROC curve of five classification models established using different ML algorithms.

The classification accuracy of feature group B was higher than that of feature group A. A maximum classification accuracy of 92.6% was achieved with the SVM classifier by applying feature group B. Meanwhile, sensitivity, specificity, and AUC were 0.850, 0.954, and 0.969, respectively. Detailed results are shown in the Table 1 and Figure 7.

**Table 1:** Performance of five algorithms with two feature groups.

**Algorithm**	**Feature**	**AUC**	**95%CI**	**Accuracy**	**Sensitivity**	**Specificity**
RF	Group A	0.897	0.894–0.900	0.824	0.518	0.934
	Group B	0.952	0.950–0.954	0.908	0.733	0.971
KNN	Group A	0.584	0.579–0.588	0.759	0.227	0.950
	Group B	0.881	0.879–0.883	0.873	0.572	0.980
XGB	Group A	0.892	0.889–0.895	0.839	0.596	0.926
	Group B	0.950	0.948–0.952	0.900	0.757	0.952
SVM	Group A	0.925	0.922–0.928	0.860	0.683	0.923
	Group B	0.969	0.967–0.970	0.926	0.850	0.954
LightGBM	Group A	0.890	0.887–0.893	0.829	0.563	0.924
	Group B	0.965	0.963–0.967	0.917	0.783	0.966

Since the overall distribution of these results was not normal, the Wilcoxon signed rank test was chosen to compare the results of the two models. The *p*-value calculated was all <0.05, so the effectiveness of feature group B was significantly better than that of feature group A.

## Discussion

Since the mechanisms of IA growth and rupture and their interactions regarding pathogenesis are not fully understood, ML(DL) may be a promising approach for risk assessment of IA growth and rupture, as it can be employed as gray or black box model. These types of models cannot be a substitute for basic research to explore the mechanisms of IA growth and rupture, but can support clinical practice and assist clinical decision-making. Morphological variables and hemodynamic parameters of IA growth were found to be correlated with the rupture risk of IAs, which are the two main risk assessment parameters for rupture. However, morphological variables and hemodynamic parameters of IA growth have different weights for rupture risk assessment, the most existing studies applied ML algorithms to morphological variables and hemodynamic parameters to assess the rupture risk of IAs (13–15, 27–30). Although the models worked with relatively high classification accuracy, how to accurately assess the rupture risk of IAs remains a challenge in clinical decision-making. The existing studies did not take the hemodynamic cloud features into consideration. In addition, the hemodynamic clouds represent the spatial distribution characteristics of hemodynamic. Compared with hemodynamic parameters, hemodynamic clouds will provide different types of discriminative features for the rupture risk assessment of IAs. Different from the existing methods, based on morphological variables and hemodynamic parameters, we added hemodynamic cloud features to improve classification performance. Our integrated model combined of ML and DL algorithms. ML algorithms such as RF, KNN, XGB, SVM, and LightGBM were, respectively, adopted to classify ruptured and unruptured IAs. In addition, DL algorithm such as Pointnet was applied to extract hemodynamic cloud features for the ML classification models, and the hemodynamic cloud features within impingement zone and inflow jet were selected. It was found that there were significant differences in the impingement zone and inflow jet between unruptured IAs and ruptured IAs. However, there are no quantified parameters associated with impingement zone and inflow jet for rupture risk assessment of IAs. It is generally accepted that complex flow patterns, small impingement regions, and narrow inflow jet are more likely to be identified in ruptured IAs (14, 21). We adopted hemodynamic cloud features within impingement zone and inflow jet to characterize impingement zone and inflow jet. In our experiment, adding consideration of hemodynamic cloud features within impingement zone and inflow jet, the classification performance is significantly improved. We found that the hemodynamic cloud features could improve the classification accuracy and AUC of the five classifier models to varying degrees. Specifically, the sensitivity of each model was improved most significantly. One possible reason is that the impingement zone and inflow jet are very specific for ruptured IAs. It should be noted that impingement zone and inflow jet are not the direct cause of IA growth rupture. It is difficult to distinguish unruptured IAs from ruptured IAs only using the characteristic parameters of impact zone and inflow jet.

In addition, impingement zone is almost a qualitative concept without quantitative criteria, and impingement zone can generally be roughly determined according to the high WSS distribution. In this study, WSS that exceeds 80% of the maximum WSS is considered as high WSS. According to the distribution of high WSS, impingement zone can be determined. The size of impact domain has a certain influence on classification performance. Furthermore, the flow rate will affect the size and location of jet impingement zone, but the impact on classification performance is acceptable. Due to the limitations of medical ethics, the patient-specific blood flow velocity is very difficult to be obtained, many studies adopted the flow velocity of a healthy subject for numerical simulation. In this study, the flow velocity was not patient-specific and taken from transcranial Doppler (TCD) performed in the craniocervical arteries of a healthy subject. Then, a flow velocity curve within a cardiac cycle (0.8 s) could be achieved through a non-linear fitting method.

In general, ML algorithms are the effective tools for processing heterogeneous data, which is relatively simple, practical, and easy to interpret. ML algorithms are relatively well-suited to identify the rupture risk of IAs. Among ML algorithms, SVM is especially suitable for classification with the relatively small sample size and the unbalanced categories as well as heterogeneous data. In our experiments, excluding hemodynamic cloud features, SVM has the best performance with accuracy of 0.860 and AUC of 0.925. Including hemodynamic cloud features, SVM still has the best performance with accuracy of 0.926 and AUC of 0.969. Our experiment results indicated that the hemodynamic cloud features within impingement zone and inflow jet significantly improve the performance of our classification models, no matter which classification model, the hemodynamic cloud features could contribute to the discrimination of ruptured IAs.

There were some limitations in this study. First, the sample size was relatively small, and more subjects are required to improve generalization performance and increase model robustness, allowing models to have more sufficient quality. Second, classification models were developed based on the data from a single institution, multicenter data can be pooled to improve the reliability of results. Third, CFD simulates fluid flows and analyzes the flow characteristics using numerical methods, in which blood was assumed to be Newtonian fluid, elasticity of the arterial wall was not considered, and the boundary conditions were not specific to the patient. Fourth, CFD simulations are time-consuming and laborious for many radiologists, neurosurgeons, and technologists, and it is not easy to perform CFD analysis in clinical practice. Finally, the findings in this study should be confirmed in a prospective study.

## Conclusion

The integrated model combining ML and DL algorithms could work with relatively high classification accuracy in assessment of the rupture risk of IAs, and the hemodynamic cloud features within impingement zone and inflow jet could significantly improve the performance of the classification models, which were important for the discrimination of ruptured IAs. Adding consideration of hemodynamic cloud features, SVM had the best classification performance with accuracy of 0.926 and AUC of 0.969.

## Data Availability Statement

The raw data supporting the conclusions of this article will be made available by the authors, without undue reservation.

## Ethics Statement

The studies involving human participants were reviewed and approved by Ethics Committee of Beijing Tiantan Hospital Affiliated to Capital Medical University. The patients/participants provided their written informed consent to participate in this study.

## Author Contributions

RC original concept and analyzed data. HL directed the research. RC, XM, ZC, and PF performed experiments. RC and HL wrote the paper. HL, RC, XM, ZC, and PF revised the paper. All authors contributed to the article and approved the submitted version.

## Funding

This study was funded by the Beijing Natural Science Foundation (No. L192044).

## Conflict of Interest

The authors declare that the research was conducted in the absence of any commercial or financial relationships that could be construed as a potential conflict of interest.

## Publisher's Note

All claims expressed in this article are solely those of the authors and do not necessarily represent those of their affiliated organizations, or those of the publisher, the editors and the reviewers. Any product that may be evaluated in this article, or claim that may be made by its manufacturer, is not guaranteed or endorsed by the publisher.
